# The causal involvement of the visual cortex in visual working memory remains uncertain

**DOI:** 10.1098/rsos.231884

**Published:** 2024-06-12

**Authors:** Pablo Rodrigo Grassi, Michael M. Bannert, Andreas Bartels

**Affiliations:** ^1^ Department of Psychology, University of Tübingen, Tübingen, Baden-Württemberg, Germany; ^2^ Centre for Integrative Neuroscience, Tübingen, Germany; ^3^ Department for High-Field Magnetic Resonance, Max Planck Institute for Biological Cybernetics, Tübingen, Germany

**Keywords:** visual working memory, sensory recruitment, TMS, visual cortex, transcranial magnetic stimulation

## Abstract

The role of the early visual cortex in visual working memory (VWM) is a matter of current debate. Neuroimaging studies have consistently shown that visual areas encode the content of working memory, while transcranial magnetic stimulation (TMS) studies have presented incongruent results. Thus, we lack conclusive evidence supporting the causal role of early visual areas in VWM. In a recent registered report, Phylactou *et al*. (Phylactou P, Shimi A, Konstantinou N 2023 *R. Soc. Open Sci*. 10, 230321 (doi:10.1098/rsos.230321)) sought to tackle this controversy via two well-powered TMS experiments, designed to correct possible methodological issues of previous attempts identified in a preceding systematic review and meta-analysis (Phylactou P, Traikapi A, Papadatou-Pastou M, Konstantinou N 2022 *Psychon. Bull. Rev.* 29, 1594–1624 (doi:10.3758/s13423-022-02107-y)). However, a key part of their critique and experimental design was based on a misunderstanding of the visual system. They disregarded two important anatomical facts, namely that early visual areas of each hemisphere represent the contralateral visual hemifield, and that each hemisphere receives equally strong input from each eye—both leading to confounded conditions and artefactual effects in their studies. Here, we explain the correct anatomy, describe why their experiments failed to address current issues in the literature and perform a thorough reanalysis of their TMS data revealing important null results. We conclude that the causal role of the visual cortex in VWM remains uncertain.

## 1. Introduction

The role of the early visual cortex in visual working memory (VWM) is a matter of current debate [1,2]. Neuroimaging research has consistently shown that early visual areas (i.e. V1, V2 and V3) represent the content of VWM (e.g. [3,4]). However, we lack conclusive evidence supporting the causal role of sensory areas in VWM, not least because previous transcranial magnetic stimulation (TMS) studies provided incongruent results (e.g. [5–11]). Some TMS studies stimulating early visual cortex (EVC) during the retention time of VWM tasks found an increase in visual working memory performance [6,9,11], while others observed a decrease [10] or had mixed results [5,7,8] (i.e. showing mixed effects or a decrease only when stimulation was applied relatively early into the retention time). A clever experimental approach used by several studies [5–8] was to present stimuli lateralized to one visual hemifield and to apply TMS contralaterally over early visual areas of one hemisphere and compare effects to an ipsilateral control condition. The rationale behind this approach was that contralateral TMS specifically targeted the part of the visual cortex involved in the visual processing, encoding and storage of visual stimuli, while ipsilateral TMS provided an ideal control condition for non-neural TMS effects such as discomfort, skin sensation and sound. This approach relies on both: the retinotopic organization of the early visual cortex in that lateralized stimuli are processed in the contralateral hemisphere, and on prior neuroimaging evidence that working memory representations are retinotopically specific (e.g. [12]).

In two recent publications, Phylactou and colleagues sought to tackle the incongruent findings regarding the causal involvement of EVC in VWM. First, via a systematic review and meta-analysis of previous TMS studies [13], and second, in a registered report involving two well-powered TMS experiments [14]. They specifically designed their TMS experiments to correct for—in their perspective—methodological issues of previous attempts, identified with the preceding meta-analysis. Among other points, they argued that previous TMS experiments might have produced inconclusive results on two grounds: first, because of recent evidence suggesting that working memory representations might also occur in the ipsilateral early visual cortex [15]; and second, because in studies using lateralized stimuli, visual information might have been processed in both hemispheres. Their second argument was based on the neuroanatomically wrong assumption that the processing of stimuli presented left or right from fixation ‘[…] does not accurately correspond to the contralateral sensory visual cortex, and could in fact be processed by the ipsilateral cortex if presented within 15° of visual angle from midline’ [14, p. 3]. They conclude that ‘[…] some TMS effects can be falsely interpreted or remain undetectable (e.g. if information processing happens in both hemispheres despite the contralateral and ipsilateral conditions […])’ [14, p. 3].

Unfortunately, this conclusion was based on a misunderstanding of the visual pathway, and it also misguided their experimental attempts to address current issues in the literature. In the main meta-analyses of their recent systematic review [13], they hypothesized that the experimental and control conditions might have been wrongly assigned in studies using ipsilateral versus contralateral stimulation, and decided to use absolute values of effect sizes instead of the more appropriate signed effects (i.e. investigating the direction of TMS effects). Similarly, in their registered TMS experiments, a key part of their experimental design (monocular presentation of central stimuli) was based on the wrong anatomical assumption that a given eye projects input from the centre of gaze (i.e. from the central retina) primarily to one hemisphere, which led to confounded conditions. They hence disregarded two important anatomical facts in their studies, namely that early visual areas of each hemisphere represent the contralateral visual hemifield, and that each hemisphere receives equally strong input from each eye.

Their misunderstanding of the visual pathway hence affected their experiments and key conclusions, as some of the TMS affects the authors’ report are artefactual instead of evidence for the involvement of early visual cortex in visual working memory. Still, while inadvertently, the authors present intriguing and noteworthy (artefactual) TMS effects that could inform future TMS experiments. However, they require contextualization and due amendment to correctly interpret their results. For these reasons, we contacted the authors with our concerns with the intention of submitting a commentary on their TMS experiment. Following an editor’s suggestion, and given the availability of the data, we deemed it appropriate to submit our view and concerns in this replication article and to present a complete analysis of the data.

In the following, we first describe two important anatomical principles of the visual pathway, describe the misguided assumptions of Phylactou *et al*., and show how this misunderstanding affected the research rationale and conclusions, in both, a meta-analysis [13] and subsequent TMS experiments [14] (§2). We show why the purported effects the authors report are confounded and artefactual and cannot be seen as evidence for the role of the early visual cortex in visual working memory. We then perform a series of reanalyses on their publicly available TMS data to offer tentative explanations for their artefactual results (§3). We conclude with some more general remarks and suggestions for future experiments (§4).

## 2. A misunderstanding of visual processing

### 2.1. Anatomy of the visual pathway

Two organizational principles of the visual pathway are relevant to understand why the meta-analysis [13] and subsequent TMS study [14] were confounded.

**Figure 1.**
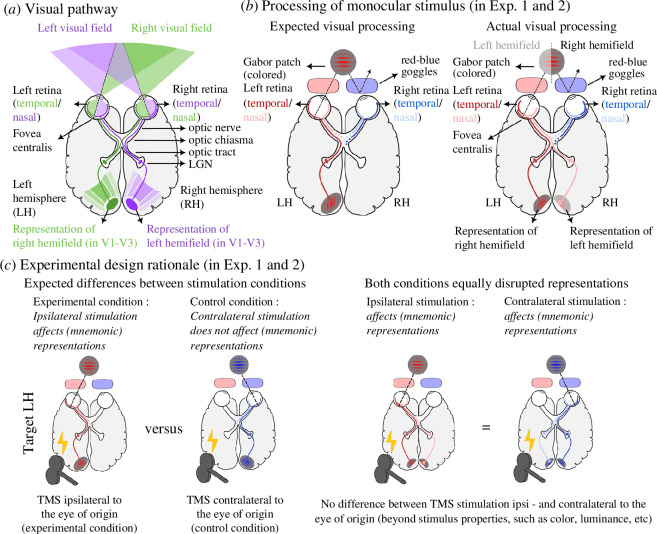
(*a*) Simplified depiction of the human visual pathway. Information from each side of the visual field is projected from the optic nerve through the optic chiasma (where the optic nerves cross) via the optic tract (continuation of the optic nerve after the chiasma) to the contralateral lateral geniculate nucleus (LGN) and from there to the contralateral visual cortex (V1 and from there to V2 and V3, etc.). Accordingly, early visual areas (V1–V3) represent the contralateral half of the visual field (e.g. left V1–V3 represent the right hemifield). At the optic chiasma, fibres from the nasal retinae cross to the contralateral tract, while fibres from the temporal retinae remain uncrossed. For example, the left temporal retina projects information of the right visual field (green) to the ipsilateral left hemisphere (LH), while the left nasal retina projects information of the left visual field (violet) to the contralateral right hemisphere (RH). Importantly, the vertical meridian (and division of projections) goes through the centre of gaze (fovea) and not through the optic disc (which is located nasally from the fovea). *(b*) Simplified visualization of the processing of dichoptically presented stimuli. Shown is a red stimulus presented at fixation and viewed through red-blue dichoptic goggles, hence selectively reaching the left eye (monocular stimulation). Phylactou *et al*. [14] wrongly assumed that a monocularly presented stimulus at fixation would initially reach the ipsilateral hemisphere (as depicted in the left panel). Instead, the left retina projects signals (via the optic nerve, tract and LGN) bilaterally to the left and right visual cortices. The left temporal retina projects information of the right visual field (right of the dotted line) to the ipsilateral hemisphere (LH; dark red), while the left nasal retina projects information from the left visual field (left of the dotted line, shown lighter for visualization) to the contralateral hemisphere (RH; light red). Accordingly, any monocular visual stimulus at fixation is actually represented in largely similar patches of early visual areas in both hemispheres (provided participants fixated correctly). (*c*) Under the mistaken assumption that a monocular stimulus presentation would be processed ipsilaterally, Phylactou *et al*. [14] presented coloured stimuli at fixation that participants viewed with red-blue goggles. As they expected to thereby separate the (initial) processing of the visual stimuli into hemispheres (shown in the left panel), they compared behavioural responses during ipsilateral (experimental condition) and contralateral (control condition) TMS stimulation. However, with regard to brain stimulation, both conditions were actually identical (shown in the right panel), as TMS in either condition would affect visual processing.

The first principle is that early visual areas of each hemisphere represent the contralateral visual hemifield (i.e. left or right half of the visual field). Each eye sends visual signals to the visual cortex of both hemispheres after crossing the optic chiasm, with information of each visual hemifield sent to the contralateral hemisphere: the left hemisphere receives input from the right visual hemifield, and the right hemisphere receives input from the left visual hemifield (see figure 1*a*). Importantly, anatomy, electrophysiology, as well as high-resolution human imaging show that the left–right split runs exactly through the fovea (small area of the central retina responsible for sharp central vision) so that visual regions in a given hemisphere corresponding to the central fovea represent the contralateral visual field [16]. Thus, even when a stimulus is viewed monocularly (i.e. if we close one eye or use red–blue goggles and view red or blue stimuli as in [14]), visual information is sent evenly to both hemispheres (left hemifield to right hemisphere and vice versa). Exceptions to this are rare cases of achiasma (lack of nerve crossing) [17], chiasm hypoplasia (crossing is less than 50%) and albinism (crossing is more than 50%) [18].

The second principle is that input from each eye is equally represented in most of the human primary visual cortex. The human primary visual cortex is organized in ocular dominance columns that process preferentially input from one or the other eye. The ocular dominance columns of each eye are equally represented from the representation of the fovea outwards up to an eccentricity of about 15°, where the widths of the columns of the ipsilateral eye begin to diminish towards the monocular crescent [19].^1^ Accordingly, evidence from anatomy, electrophysiology and neuroimaging points to a balanced contribution of the ipsilateral and contralateral eye to the human primary visual cortex of each hemisphere [20].

### 2.2. A misguided critique of previous TMS experiments

In their systematic review and meta-analysis [13], Phylactou and colleagues argue that previous TMS experiments presenting lateralized stimuli and comparing ipsilateral versus contralateral stimulation [5–8] might have led to incongruent results, ‘[…] because of the lack of dichoptic stimulus presentation (see e.g. [21]), it remains possible that encoding of the visual information was in fact processed by the sensory visual cortex in both hemispheres ([22, 15]) and thus the effect remained undetected when comparing ipsilateral versus the contralateral condition […]’ ([13], p. 1606). On the same grounds, they argue that the direction of the effects might be often falsely interpreted (i.e. inhibition as facilitation or vice versa) and that because of ‘[…] recent evidence supporting the role of the ipsilateral sensory visual cortex in visual processing ([15]) and the visual pathway neuroanatomy (see [23,24]), it could be reasonable to assume that the ipsilateral sensory visual cortex is in fact the experimental condition’ ([13], p. 1599). They conclude with the suggestion that ‘[…] future work could aim to explore the contradictory effects reported between the studies, by investigating the role of the sensory visual cortex in VSTM, while presenting stimuli monocularly (e.g., [21]) […]’ ([13], p. 1618). Their argument is therefore based on two key assumptions: (A) an assumption of the spatial specificity of WM, based on recent imaging evidence (from Zhao *et al*., [15]) that both the ipsilateral and contralateral V1 and V2 represent feature-based working memory (i.e. grating orientations) [15] and (B) a neuroanatomical assumption that previously used stimuli might have been processed by both hemispheres and that only monocular presentation can ensure unilateral cortical processing.

Regarding the assumption of *spatial specificity of WM* (A), it should be noted that while there is some neuroimaging evidence that feature-based visual working memory can also involve the ipsilateral hemisphere [15,25], it has been consistently shown that when spatial information is task-relevant (as was the case in many of the prior TMS experiments), working memory representations are spatially specific [12,26–28]. Moreover, even if working memory representations were present in both contralateral and ipsilateral visual areas, we do not see how either hemisphere could then be unmistakably assigned to be the experimental condition (as they did in their subsequent TMS experiment, see below).

In turn, and more importantly, the *neuroanatomical* assumption (B) is based on a misunderstanding of the anatomy of the visual pathway. Phylactou and colleagues wrongly assumed that stimuli presented within 15° of the visual angle (as used in the prior TMS experiments, [5–8]) would be processed by both hemispheres if viewed with both eyes. For example, in their TMS study, they write: ‘[…] considering the neuroanatomy of the visual pathway system, the binocular presentation of stimuli either left or right close to the midline of the visual field—as was the case in the majority of the previous studies—does not accurately correspond to the contralateral sensory visual cortex, and could in fact be processed by the ipsilateral cortex if presented within 15° of visual angle from midline [23,24]’ ([14], p. 3). Accordingly, they suggest presenting stimuli monocularly. However, as described above and contrary to their assumption, information on each visual hemifield is projected separately to early visual areas (V1–V3) of the contralateral hemisphere (see figure 1*a*). Presenting stimuli monocularly or binocularly will not affect in which hemisphere they are processed—input from both eyes is equally represented in a given hemisphere for central vision (from 0° to about 15° visual degrees), such that the left primary visual cortex receives input from the right visual hemifield, equally from left and right eye, and the right primary visual cortex receives input from the left visual hemifield, equally from left and right eye. Thus, lateralized stimuli viewed with both eyes in previous TMS experiments were in fact correctly processed in the contralateral early visual areas only.^2^

### 2.3. Consequence for the meta-analysis

In their systematic review and meta-analysis, Phylactou and colleagues investigated the effect of TMS on VWM performance via two main meta-analyses. The first meta-analysis investigated the effect of early TMS on VWM performance (<200 ms, ‘encoding phase’). The second investigated the effect of late TMS (>200 ms, ‘maintenance phase’). Following the rationale of their critique (outlined above), the authors decided to use absolute values of the effect sizes, instead of investigating the direction of TMS effects (improvement or decrease of VWM performances). In other words, the authors ignored whether TMS led to weakening or strengthening of VWM performance as a function of ipsilateral or contralateral stimulation, by always taking the absolute (positively signed) effect into their meta-analysis. Accordingly, all effect sizes were forced to be positive, leading to an artificially strong effect that was significantly different from zero in both meta-analyses (TMS during encoding and maintenance phases of VWM tasks). In contrast, subsequent meta-analyses on signed effect sizes (the authors deemed as exploratory) revealed much weaker effect sizes and in opposite directions (TMS during the encoding phase of working memory items decreased performance, while TMS during maintenance of working memory items improved performance).^3^ Hence, the analysis of the encoding and maintenance phases together yielded no significant effect. Yet, their main meta-analysis yielded an artificially inflated estimate of TMS effect sizes and thus presented an overly optimistic and biased picture of the causal role of EVC in VWM.

### 2.4. Two TMS experiments with surprising results

Following the rationale outlined in their systematic review, Phylactou and colleagues sought to tackle the mistakenly identified methodological issues of previous TMS experiments via a registered report involving two well-powered TMS experiments [14]. In line with their suggestion for future experiments, their TMS experiments aimed to separate the initial processing of visual information into hemispheres by presenting their stimuli monocularly (yet centrally at fixation): participants viewed centrally presented coloured stimuli through red-blue goggles so that red stimuli were only seen with the left eye and blue stimuli with the right eye. Participants were shown either a red or a blue Gabor patch at fixation and after a short retention time (2 s) asked if a test probe had the same orientation as the one memorized. TMS was applied over the left or right occipital cortex during the retention time (at different time points). Depending on the eye of presentation, left or right occipital cortex served (mistakenly) as experimental versus control conditions. In the first experiment, TMS was applied at 0, 0.2 or 1 s into the retention time. In the second experiment, TMS was applied at 0.2 or 1 s, and an additional sham stimulation condition using a dedicated coil was included. The sham condition was correctly introduced to control for the possibility that occipital TMS affects both hemispheres, to aid the interpretation of the direction of effects (inhibitory versus facilitatory) and, most importantly, given the possibility the stimuli would be processed in both hemispheres.

However, the approach by Phylactou and colleagues to target the site of visual cortex processing visual information was based on the mistaken assumption that monocular visual information presented at fixation ‘will initially be processed solely by the ipsilateral (to the eye receiving the information) sensory visual cortex’ ([14], p.5). They wrongly assumed that the centrally presented red patch (monocularly viewed by the left eye) would be processed only in the ipsilateral left hemisphere and the blue patch only in the right hemisphere (see figure 1*b*, left panel). Instead, the visual stimuli were equally processed by both hemispheres (as shown in figure 1*c*, right panel). Accordingly, they analysed their data by comparing ‘ipsilateral’ (experimental condition) versus ‘contralateral’ (control condition) stimulation and expected—following their rationale—to observe a difference of performance (*d*′) following ipsilateral TMS stimulation, i.e. performance in trials showing red patches should be different to those using blue patches during left occipital TMS stimulation and vice versa (see figure 1*c*, left panel). Moreover, they additionally tested for overall differences between real TMS and sham TMS (independent of the stimulation side) in their second experiment. The validity of the comparison between ‘real TMS’ and ‘sham TMS’ conditions was not compromised by the incorrect anatomical assumptions.

Phylactou and colleagues observed all of their expected results. First, and to our surprise, they systematically observed a decrease in performance following ipsilateral compared with contralateral TMS stimulation, in both experiments. Second, and more expectedly, they showed a decrease in performance when comparing real TMS with sham stimulation (in the second experiment).

### 2.5. Consequence of the TMS results

As described above, any monocular stimulus presented at the centre of gaze will be represented in a roughly equally large surface of the early visual cortex of both hemispheres [19]. Hence, the ‘ipsilateral’ and ‘contralateral’ conditions in the aforementioned TMS experiments did not separate input or processing to one or the other hemisphere: both conditions led to equal processing in both visual hemispheres (see figure 1*b*, right panel). Importantly, this still holds true even if the central representations were to be double-represented by overlapping projections to each hemisphere (which is a matter of debate, e.g. [31–33]). The debate about the potential foveal double-representation hence does not affect the above reasoning. In terms of targeted neural populations by TMS, there was no difference whatsoever between the ‘ipsilateral’ and ‘contralateral’ conditions: in both conditions, neural populations of both eyes were equally affected (see figure 1*c*, right panel). TMS applied to any hemisphere would disrupt the processing of both red and blue stimuli equally. In consequence, and in contrast to the authors’ conclusions, the reported differences between ‘ipsilateral’ and ‘contralateral’ responses in these experiments cannot and do not reveal any evidence for a causal role of early visual areas in VWM. Only the comparison between real TMS and sham TMS of their second experiment potentially provides evidence for an involvement of EVC in VWM, as these tests were not directly affected by the confounded stimulation sides.

## 3. Reanalysis of TMS data: tentative explanations for the (artefactual) effects

In §2, we described that the approach of monocular yet central stimulus presentation as an attempt to isolate visual stimulation to one hemisphere in the TMS experiments conducted by Phylactou *et al*. [14] was based on a misunderstanding of visual cortex organization. And yet, the observed effect when comparing ipsilateral and contralateral TMS in their experiments appeared to be consistent with the authors’ reasoning. They observed a robust decrease in performance following occipital TMS stimulation of the ipsilateral hemisphere (to the eye of origin) compared to contralateral stimulation. How is this possible? What do these lateralized TMS effects show, and how can we interpret them?

To address these questions, we performed a series of reanalyses using the publicly available data from the TMS experiments [14]. We replicated their registered analyses (H1–H7), presented reanalyses including excluded participants and, importantly, performed additional analyses on the data from the second experiment that took into account their control sham condition. Our additional analyses revealed that the experiments did indeed provide the (expected) null finding of the effect of TMS stimulation side.

### 3.1. Material and methods

Here, we provide a brief description of the paradigm and experiment of Phylactou *et al*. [14], followed by more detailed descriptions of our additional analyses using their publicly available data. The paradigm used in the TMS experiments was a delayed change-detection task. Participants wearing red-blue goggles were shown a coloured Gabor patch (either red or blue; diameter: 1° of visual angle) at fixation for 100 ms and, after a short retention time (2 s), asked if a test probe had the same orientation as the one memorized. TMS double-pulses were applied during the retention time. In the first experiment (Exp. 1), pulses were applied 0, 0.2 or 1 s after the memory stimulus onset over the left or right occipital cortex (counterbalanced across participants). In the second experiment (Exp. 2), an additional sham stimulation condition using a dedicated coil was introduced and stimulation was applied at 0.2 or 1 s. The dependent variable in both experiments was the detection sensitivity *d′*.

Data for the reanalyses can be found in the public repository of the original study (https://royalsocietypublishing.org/doi/suppl/10.1098/rsos.230321). Following their analysis procedure, we performed a series of Bayesian paired *t*-tests using the same priors. Bayesian analyses were performed using the Python toolbox pingouin [34] and were based on their provided analysis scripts for comparison. For further details about experimental design, behavioural paradigm, TMS stimulation protocol and analysis, please see the original study [14].

#### 3.1.1. Participants

The first experiment had a total of 43 participants, seven of whom were excluded from the original analysis (remaining *n* = 36). Four participants were excluded because their accuracy was below 60% (as preregistered). Three further participants were excluded due to self-reported vision problems (reported during study debriefing). One had a self-reported uncorrected astigmatism, and two had self-reported amblyopia. The second experiment had a total of 32 participants, four of whom were excluded from the original analysis (remaining *n* = 28). Three participants were excluded because of accuracy below 60% and one due to self-reported history of amblyopia.

#### 3.1.2. Reanalysis procedure

In their TMS study, Phylactou and colleagues report seven preregistered analyses (three for Exp. 1 and four for Exp. 2) [14]. For the first experiment, H1 to H3 compared performance between ipsilateral and contralateral stimulation during three TMS stimulation time points (0, 0.2 and 1 s, respectively). Similarly, for the second experiment, H4 and H6 tested for differences between ipsilateral and contralateral stimulation for early (0.2 s) and late (1 s) TMS stimulation. Finally, differences between real TMS and sham TMS were analysed for early (H5, 0.2 s) and late (H7, 1 s) stimulation. The authors consistently reported that performance (*d′*) during occipital TMS stimulation of the hemisphere ipsilateral to the eye of origin was worse compared with contralateral stimulation, regardless of stimulation time (in H1, H2, H3, H4 and H6). They further show that performance following real TMS was worse compared with sham TMS at both stimulation times (in H5 and H7). In view of the intriguing results showing an effect of TMS stimulation side (ipsilateral versus contralateral), we decided to perform a series of dedicated reanalyses.

First, we made sure to reproduce the results for each of the registered tests as reported in their manuscript (H1–H7). Thanks to their transparent approach and openly available data and code, the reproduction of their results was successful.

Second, we investigated the responses following sham TMS stimulation in detail. In particular, we tested for differences between the ipsilateral and contralateral conditions during sham stimulation (i.e. trials in which no real TMS pulse was applied). We additionally tested for an effect of stimulation side (ipsilateral versus contralateral) after subtracting the corresponding sham-TMS values from the real-TMS values (sham-corrected TMS), which is common practice in TMS experiments. Using the sham TMS trials as a control baseline allows to control for (some) confounding factors accompanying the intended neural effects following TMS stimulation (like noise, pressure on the scalp, etc.). If the purported effects (i.e. a decrease in performance after ipsilateral TMS stimulation) were to be observed in the sham TMS trials, it means that the effect cannot be unequivocally attributable to a TMS disruption of visual working memory. None of these tests were reported in the original study.

Moreover, we tested for differences between the ipsilateral and contralateral conditions for participants in whom TMS was applied over the left and right hemispheres separately.

Finally, we repeated the tests after inclusion of the participants who had been excluded based on self-reported eye problems and after inclusion of all participants (i.e. also including those with an accuracy below 60%). We decided to perform these exploratory analyses—in the context of the unexpected effects—to test the robustness of the results and investigate how the exclusion of participants due to self-reported eye problems after the experiment affected the results. All excluded participants of Exp. 1 had an accuracy above 0.56 (group mean accuracy without exclusions was 0.71 ± 0.08 s.d.). Similarly, excluded participants from Exp. 2 performed above 0.57 (group mean accuracy without exclusions was 0.7 ± 0.07 s.d.).

### 3.2. Results

First, we replicated all of the original tests, showing a consistent decrease in performance following ipsilateral stimulation when compared with contralateral stimulation across different time points in both experiments (Exp. 1: H1, H2, H3; Exp. 2: H4 and H6). Equally, we replicated the decrease in performance following real TMS stimulation when compared with sham stimulation (averaged across ipsilateral and contralateral conditions) (Exp. 2: H5 and H7) (results listed in table 1).

**Table 1.** Bayesian paired *t*-tests results. The first column presents results using the same sample of participants as in the original study (Exp. 1: *n* = 36; Exp. 2: *n* = 28). The second column presents the results after only excluding subjects based on performance (as registered), i.e. after inclusion of participants excluded because of self-reported eye-problems (Exp. 1: *n* = 39; Exp. 2: *n* = 29). The third column shows test results without any exclusion (Exp. 1: *n* = 43; Exp. 2: *n* = 32). Bayes Factors in italics show novel analyses. Bayes factors in bold show a decrease in evidence in comparison to the original analysis.

Bayesian paired *t*-tests	BF_10_	exclusion: **as registered**	**no exclusion**
**reanalysis of registered tests**			
exp. 1: ipsi versus contra at 0 s (H1)	29.4	***1.95***	***2.95***
exp. 1: ipsi versus contra at 0.2 s (H2)	35.99	***1.9***	*3.07*
exp. 1: ipsi versus contra at 1 s (H3)	3.67	***0.79***	***0.66***
exp. 2: ipsi versus contra at 0.2 s (H4)	288.2	*225.5*	*356.3*
exp. 2: TMS versus sham at 0.2 s (H5)	7.75	*4.05*	***1.99***
exp. 2: ipsi versus contra at 1 s (H6)	15.5	*20.9*	*33.1*
exp. 2: TMS versus sham at 1 s (H7)	8.39	12.35	35.35
**effect of side in sham TMS (exp. 2)**			
Sham TMS at 0.2 s (ipsi versus contra)	*16.65*	*30.43*	*73.64*
Sham TMS at 1 s (ipsi versus contra)	*3.3*	*4.04*	*6.42*
**sham-corrected effect of side (exp. 2)**			
TMS at 0.2 s (ipsi versus contra)	*0.67*	*0.29*	*0.24*
TMS at 1 s (ipsi versus contra)	*0.28*	*0.27*	*0.23*

**Figure 2.**
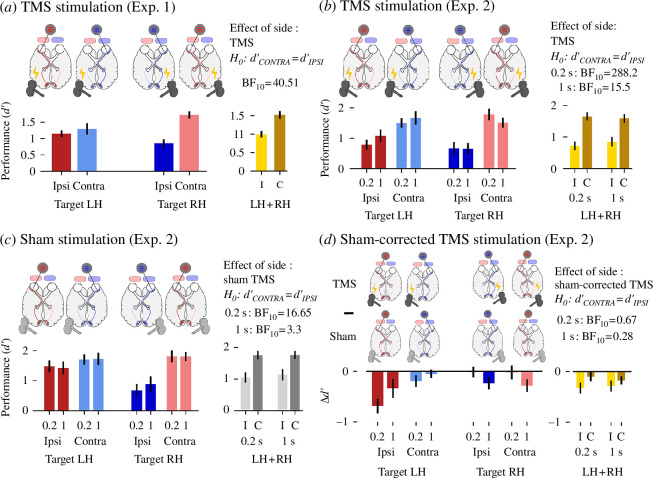
(*a*) In the first experiment, TMS applied to the visual cortex ipsilateral to the eye of origin (ipsi, *i*) decreased performance in a change-delay task compared with contralateral stimulation (contra, *c*) at all tested time points (0, 0.2 and 1 s). Bar plot on the left shows the effect of TMS on behavioural performance (*d’*) separated by stimulation side (ipsilateral and contralateral) and stimulated hemisphere (target LH and target RH), pooled across times (0, 0.2 and 1 s). Bar plot on the right shows the effect of stimulation side across time points for all participants (LH + RH). Note that the effect of stimulation side was evident only in participants in whom TMS was applied over the right hemisphere (target RH). (*b*) In the second experiment, occipital TMS stimulation also decreased WM performance when applied ipsilaterally to the eye of origin at both time points (0.2 and 1 s). (*c*) Similarly, sham stimulation revealed the same lateralized effect, at both time points (0.2 and 1 s). As in Exp 1., the effect was only visible when targeting the right hemisphere (RH). (*d*) Subtracting sham stimulation from the corresponding experimental conditions and testing for a difference between contralateral and ipsilateral stimulation revealed evidence for an absence of an effect (BF_10_ < 1) at both time points (0.2 and 1 s, see right bar plots).

Perhaps the most important new analysis we performed concerned sham TMS: we tested the effect of stimulation side (ipsilateral versus contralateral) in the sham condition of Exp. 2 (i.e. trials without any real TMS stimulation). We observed that even sham stimulation over the hemisphere ipsilateral to the eye receiving stimulus input impaired performance when compared with contralateral stimulation, at both time points (0.2 s: BF_10_ = 16.65; 1 s: BF_10_ = 3.3; see our reanalysis in figure 2*c,d* and table 1). Moreover, when using the corresponding sham condition as a control baseline (i.e. subtracting it from the experimental condition), there was no effect of the stimulation side (ipsilateral versus contralateral) at either time point (sham-corrected TMS effects at 0.2 s: BF_10_ = 0.67 and 1 s: BF_10_ = 0.28, see our reanalysis in figure 2*d* and table 1). These tests provide evidence that TMS played no role in the observed differences between ipsilateral and contralateral conditions (i.e. H1–H4 and H6). Note that this finding shows that a key result of the original paper by Phylactou and colleagues was confounded.

We followed up on these findings in more detail, as the lateralization effects are nevertheless surprising, and require non-neural explanations as they were observed in TMS as well as in sham TMS. Interestingly, the effect of TMS side (ipsilateral versus contralateral) was mainly present following right visual cortex stimulation and less so following left visual cortex stimulation (visible in figure 2*a–c*). All tests of the TMS effect of side in participants with right stimulation (*n* = 19 and *n* = 15 in Exp. 1 and 2, respectively) were above a BF_10_ of 3 (Exp. 1: 0 s = 71.9, 0.2 s = 19.2, 1 s = 25.5; Exp. 2 with real TMS: 0.2 s = 24.98, 1 s = 12.6, with sham TMS: 0.2 s = 54.97, 1 s = 3.48). In contrast, most tests in participants with left stimulations (*n* = 17 and *n* = 13 in Exp. 1 and 2, respectively) had a BF_10_ below 1, revealing evidence for the null hypothesis instead (Exp. 1: 0 s = 0.35, 0.2 s = 0.68, 1 s = 0.25; Exp. 2 with real TMS: 0.2 s = 2.65, 1 s = 0.696, with sham TMS: 0.2 s = 0.36, 1 s = 0.38).

Inclusion of participants excluded in the original study reduced remarkably the disruptive effect of ipsilateral TMS stimulation in Exp. 1 (main effect of side across TMS stimulation times decreased from BF_10_ = 40.51 to BF_10_ = 2.49), but less so in Exp. 2. Similarly, the comparison between TMS and sham stimulation (regardless of TMS stimulation side) suggests that the effects were stronger later in time (BF_10_ at 0.2 s = 1.99 and at 1 s = 33.1, see table 1).

### 3.3. Discussion of TMS results

Despite the neural equivalence of ipsilateral and contralateral processing of the centrally presented stimuli, occipital TMS stimulation ipsilateral to the eye of origin systematically impaired visual working memory performance when compared with contralateral stimulation. However, the very same effect was also observed following sham stimulation (i.e. in trials without any real TMS stimulation). Hence, real TMS and sham TMS led to the same results. The differences between ipsilateral versus contralateral conditions (in both TMS and sham conditions of experiments 1 and 2, corresponding to H1–H4 and H6; shown in figure 2*a,b*, respectively) can hence not be attributed to a disruption of VWM in early visual cortex via TMS, but instead to other (unintended) factors differentially affecting the processing of monocularly viewed stimuli that occur during both TMS and sham TMS equally. Interestingly, this null finding regarding the key hypotheses remained largely unacknowledged in the original publication by Phylactou *et al*. [14], potentially due to their rigorous adherence to the analysis protocol that did not plan direct comparisons between TMS and sham in the pre-registered tests. This in turn emphasizes the importance of exploratory analyses.

What might have caused this artefactual effect in the TMS and sham conditions alike? While we can only speculate as to what drove this effect, a number of possible confounds come to mind. First, (sham-)TMS could have induced and/or conditioned (ipsilateral) blinks that differentially affected performance during ipsilateral stimulation. However, missing eye-tracking data precludes testing this possibility. Then, as coloured stimuli were not matched in terms of luminance, contrast, difficulty and presented eye, these uncontrolled differences could have systematically affected ipsilateral processing, potentially in combination with subject-dependent biases (such as eye dominance). Moreover, limitations of the sampling procedure might have further affected the results, as a reanalysis including the three participants excluded due to self-reported eye problems revealed only anecdotal evidence for the effect in the first experiment (BF_10_ < 3). Yet, sampling biases cannot explain the strong effect observed in Exp. 2. Finally, as our reanalyses show that the effect was systematically present during occipital stimulation of the right hemisphere, the observed effect could potentially be explained by unspecific effects due to coil positioning [35], possibly in combination with further factors (such as the above-mentioned stimulus differences). Importantly, and regardless of what exactly drove the effect, this unexpectedly strong artefactual effect might be relevant for future TMS experiments over the visual cortex and underscores the importance of (sham) controls.

Then, and in accordance with Phylactou *et al*.’s, conclusions [14], the observed performance decrease in TMS trials compared with sham across stimulation sides (i.e. averaged across ipsilateral and contralateral conditions) could be seen as evidence that the early visual cortex is generally involved in VWM (H5 and H7). These tests were unaffected by confounded stimulation conditions. However, a more compelling and parsimonious explanation is that, as TMS over the visual cortex was performed at 110% phosphene threshold, phosphenes were (per definition) likely induced during TMS trials. Performance in TMS trials may then have been selectively affected simply because phosphenes could have acted as a distractor (i.e. attracting attentional resources, e.g. [8]), in combination with further factors, such as differences in scalp sensation, blinks or discomfort [36]. This interpretation is supported by the stronger TMS effect when stimulation occurred later in time and thus, closer to the probe (see our reanalysis of Exp. 2).

## 4. Concluding remarks

Together, Phylactou *et al*. laudably set out to address incongruent results from previous studies in a meta-analysis [13] and in a registered report [14]. Unfortunately, the procedures in both of their studies were based on a misunderstanding of the visual system.

In their meta-analysis, Phylactou *et al*. argued that previous studies using lateralized stimuli might have produced incongruent results, because they wrongly believed stimuli could have been visually processed by both hemispheres when presented lateralized and the experimental condition could not be unambiguously assigned to either stimulation side (ipsilateral or contralateral). Therefore, they chose to perform their main analyses using unsigned absolute effect sizes from all studies (i.e. including those without lateralized stimuli), hence artificially creating strong effects. In fact, previous TMS experiments produced mixed results [5–11].

Similarly, they performed their TMS experiments under the erroneous assumption that monocular stimuli at fixation are selectively projected to the ipsilateral hemisphere and that this approach can thus be used to separate visual processing into hemispheres. In truth, their approach led to visual stimuli being processed in both hemispheres. This nulled any difference between their ipsilateral and contralateral conditions in terms of TMS. Accordingly, our analysis of their data testing the effect of stimulation side after correcting for unspecific factors related to sham stimulation revealed a null finding. We can only speculate what might have caused the intriguing decrease in performance following both ipsilateral TMS and ipsilateral sham TMS with respect to the eye of origin, yet hope our observations inform future studies. Only the second result that combined ipsilateral and contralateral conditions to compare TMS versus sham provides valid evidence. It suggests a potential late involvement of early visual areas in VWM but might require follow-up experiments to exclude possible confounds as discussed above (i.e. phosphenes, scalp sensation, blinks and discomfort).

Recent evidence from laminar electrophysiology [26] and neuroimaging [37,38] suggest that working memory representations in early visual cortex are mediated via feedback connections targeting the superficial and deeper layer of the cortex coming from higher-level areas, such as the parietal cortex [39]. Accordingly, while the sensory processing of visual working memory items is driven by incoming visual information into the middle layers of the visual cortex in a retinotopic manner (i.e. with a strict contralateral representation), feedback-mediated mnemonic representations in the visual cortex may be both, retinotopic and non-retinotopic. Similarly to how visual areas are modulated by spatial attention retinotopically [40] and by feature-based attention non-retinotopically [41], working memory representations may be retinotopically specific when the task requires maintenance of spatial information [12,26–28] or non-retinotopic (i.e. involving contralateral and ipsilateral representations), when the task may be solved by maintaining feature-based information and spatial information is task-irrelevant [15,25].

Notably, the possibility of a global, non-retinotopic involvement of the visual cortex during their experiment was one of the reasons Phylactou *et al*. [14] introduced the sham TMS condition in their second experiment. The results presented here highlight the importance of this decision: we show that ipsilateral versus contralateral effects found in the TMS conditions were also present in the sham TMS control, and only our re-analysis that combined sham and non-sham TMS showed that ipsilateral versus contralateral effects amounted to a null-finding that is consistent with the underlying anatomy. This underscores that the inclusion of dedicated control conditions, such as sham TMS, is essential to address potential unspecific effects unrelated to the neural effects of TMS stimulation (such as coil positioning, noise, etc.).

Future TMS studies examining early, retinotopically specific processing (visual–spatial WM, spatial attention, etc.) are better advised to present stimuli lateralized to one hemifield as this indeed ensures contralateral hemispheric processing. Lower visual field presentation may additionally help, as the dorsal parts of V2–V3 are the most likely targets of occipital TMS [42].

Moreover, it should be acknowledged that Phylactou *et al*. [14] strictly and transparently followed their registered report and made all stages available to the community. This highlights general points about registered reports. First, the importance of reviewers and editors at all stages of registered reports, as they could have drawn the authors’ attention to the flawed anatomical assumptions and guided their time and resources into a more fruitful direction. Second, while registered reports help address important biases in science [43], they are no insurance against inadvertent scientific mishaps, and remind us that researchers should remain inquisitive about their data—in this case for example, the inclusion of the sham TMS conditions in the main analysis could have drawn the authors attention to the null-finding, but would have required performing this analysis even though it was not pre-registered. Generally speaking, addressing publication biases and selective reporting via pre-planned analyses is no safeguard against missing important tests. Finally, the public availability of data was central for a complete analysis of the present study and underscores the importance of the transparency and data sharing that accompany registered reports.

In conclusion, in view of the null results presented here and the incongruent results of previous studies, a causal role of early visual areas in visual working memory remains uncertain [2].

## Data Availability

Codes for reanalysis and visualisations used in this article are available at [44].

## Footnotes

^1^
Note that this eccentricity (>15 visual degrees) is not relevant to any of the behavioural, TMS or fMRI studies on VWM considered here, as they all presented stimuli (even the lateralized ones) more centrally.

^2^
Please note that neither the binocular rivalry papers [25,26], nor the visual anatomy papers [27,28] the authors refer to support their misunderstanding.

^3^
It is worth noting that several of the effects seem to have been wrongly coded in their meta-analyses. For example, out of the seven studies that stimulated early visual areas during the maintenance phase of WM [7–9,12,13,29,30], five had coding problems. Effect sizes from Cattaneo *et al*. [13], Zokaei *et al*. [30](one effect), Vam Lamsweerde *et al*. [9] and Jia *et al*. [12] were coded as facilitatory (positive effect sizes), while they were in fact all inhibitory (i.e. TMS negatively affected the performance in VWM tasks). Moreover, the number of participants in two of the effects from the study from Saad and Silvanto [29] was doubled (from 8 to 16) and the effect sizes from Cattaneo *et al*., [13] were repeated and from the wrong experiment.
